# Targeting mTOR Kinase with Natural Compounds: Potent ATP-Competitive Inhibition Through Enhanced Binding Mechanisms

**DOI:** 10.3390/ph17121677

**Published:** 2024-12-12

**Authors:** Sulaiman K. Marafie, Eman Alshawaf, Fahd Al-Mulla, Jehad Abubaker, Anwar Mohammad

**Affiliations:** 1 Biochemistry and Molecular Biology Department, Dasman Diabetes Institute, Dasman 15462, Kuwait; sulaiman.marafie@dasmaninstitute.org (S.K.M.); eman.alshawaf@dasmaninstitute.org (E.A.); 2 Translational Research Department, Dasman Diabetes Institute, Dasman 15462, Kuwait; fahd.almulla@dasmaninstitute.org

**Keywords:** mTOR, kinase domain, ATP, mTOR inhibitors, natural compounds, molecular simulation

## Abstract

**Background/Objectives**: The mammalian target of the rapamycin (mTOR) signaling pathway is a central regulator of cell growth, proliferation, metabolism, and survival. Dysregulation of mTOR signaling contributes to many human diseases, including cancer, diabetes, and obesity. Therefore, inhibitors against mTOR’s catalytic kinase domain (KD) have been developed and have shown significant antitumor activities, making it a promising therapeutic target. The ATP–KD interaction is particularly important for mTOR to exert its cellular functions, and such inhibitors have demonstrated efficient attenuation of overall mTOR activity. **Methods**: In this study, we screened the Traditional Chinese Medicine (TCM) database, which enlists natural products that capture the relationships between drugs targets and diseases. Our aim was to identify potential ATP-competitive agonists that target the mTOR-KD and compete with ATP to bind the mTOR-KD serving as potential potent mTOR inhibitors. **Results**: We identified two compounds that demonstrated interatomic interactions similar to those of ATP–mTOR. The conformational stability and dynamic features of the mTOR-KD bound to the selected compounds were tested by subjecting each complex to 200 ns molecular dynamic (MD) simulations and molecular mechanics/generalized Born surface area (MM/GBSA) to extract free binding energies. We show the effectiveness of both compounds in forming stable complexes with the mTOR-KD, which is more effective than the mTOR-KD–ATP complex with more robust binding affinities. **Conclusions**: This study implies that both compounds could serve as potential therapeutic inhibitors of mTOR, regulating its function and, therefore, mitigating human disease progression.

## 1. Introduction

Signaling pathways play crucial roles in the development and progression of many human diseases. Among those is the mammalian target of the rapamycin (mTOR) signaling pathway, which is a central regulator of cell growth, proliferation, metabolism, survival, and aging. It maintains cellular homeostasis by integrating signals from nutrients, growth factors, and cellular energy levels [1,2]. Dysregulation of mTOR signaling contributes to numerous diseases, whereby the continuous activation of mTOR has been implicated in many cancers due to uncontrolled cell proliferation and growth [3,4]. Its dysregulation has also been linked to metabolic disorders such as diabetes and obesity, as well as neurodegenerative diseases and cardiovascular diseases [5,6,7,8,9]. In diabetes, mTOR is key in affecting insulin resistance and sensitivity, glucose uptake, and lipid metabolism [5]. Moreover, it has been demonstrated to have essential roles in regulating T cells, which are crucial for the immune response implicated in metabolic diseases [10].

mTOR is part of a vast network of signaling pathways that lie downstream of the phosphatidylinositol 3 kinase (PI3K) pathway and involves other key players that contribute to the development of human diseases. These include, but are not limited to, protein kinase B (Akt), serum/glucocorticoid-regulated kinase 1 (SGK-1), and AMP-activated protein kinase (AMPK) signaling, which contribute to overall mTOR functions [11,12]. mTOR functions by forming two distinct complexes, mTOR Complex 1 (mTORC1) and mTOR Complex 2 (mTORC2), which are differentially regulated. Growth factors and nutrients mainly activate mTORC1 to promote cell growth, survival, and autophagy. Alternatively, mTORC2 is also triggered by growth factors but is less sensitive to nutrients. Unlike the extensively studied mTORC1, mTORC2 is less understood but has been demonstrated to have roles in regulating the actin cytoskeleton, cell size, and glucose uptake [7,13,14,15].

mTORC1, once activated, phosphorylates its downstream targets P70-S6K1 and 2 (S6K1 and S6K2) and 4E binding proteins 1 and 2 (4E-BP1 and 4E-BP2), whereas mTORC2 activation directly phosphorylates Akt at serine 473 [16]. Despite mTORC1 and mTORC2 being differentially regulated, they can modulate each other’s activity during protein production [17]. The dysregulation of mTORC1 can promote mTORC2 activity [18], while other studies have reported the opposite, with mTORC1-mediated S6K1 phosphorylation hindering the overall mTORC2 activity [19]. The hyperactivation of insulin receptor substrate 1 (IRS-1), an important upstream regulator of mTOR, has also been linked to diabetes [6,20]. Moreover, a study by Marafie et al. demonstrated potential crosstalk between Akt and IRS-1 via mTORC2 that could affect glucose-stimulated insulin secretion (GSIS) under lipotoxic conditions [21]. One of the key inhibitors of mTOR signaling is the FDA-approved drug rapamycin. Rapamycin directly interacts with mTOR, weakening and dissociating mTORC1 and abolishing its cellular functions. It was initially believed that mTORC1, and not mTORC2, was the sole target for rapamycin. However, recent studies have demonstrated that prolonged rapamycin treatment inhibits mTORC2 signaling [22]. Therefore, understanding the mechanism of action of mTOR signaling is vital for developing targeted therapies, which can modulate its pathway to achieve cellular homeostasis and, eventually, mitigate disease progression.

Despite the two well-established mTOR complexes, recent studies have implicated the potential formation of a third mTOR complex, namely mTORC3, that is activated and regulated independently of both mTORC1 and mTORC2 [23,24,25,26,27,28]. Nguyen et al. reported that mammalian enhancer-of-akt-1-7 (mEAK-7), an evolutionarily conserved protein that is highly expressed in different types of cancer cells, interacts with both mTOR and mLST8, but does not interact with other components of mTORC1 or mTORC2. mEAK-7 is important for mTOR signaling in the lysosome and is a key regulator of cell proliferation through the activation of S6K2 and 4E-BP1 [25]. Moreover, the mEAK-7-mTOR interaction is strongly associated with non-small-cell lung carcinoma progression. This association was attributed to mEAK-7 further interacting with the DNA-dependent protein kinase catalytic subunit isoform 1 (DNA-PKcs). As a result, the mEAK-7-mediated mTOR-DNA–PKcs complex was shown to be a key regulator in human cancer cells, acting through the S6K2 axis [24]. Other studies have also demonstrated the importance of mEAK-7 complexing with mTOR and have shown that microRNA, specifically miR-19911-3p, negatively regulates mEAK-7 affecting mTOR’s lysosomal localization, and significantly decreased cell proliferation and migration. This implies the tumor-suppressive role of miR-1911-3p in modulating mTOR signaling by regulating mEAK7 in human cancers [26]. Others have also demonstrated the potential formation of mTORC3, where mTOR was shown to interact with the G-protein-coupled receptor kinase-interacting protein 1 (GIT1) and the transcription factor ETV7, independently of Raptor or Rictor in regulating tumorigenicity [27,28]. Taken together, these findings highlight that the discovery of mTORC3 represents a paradigm shift in understanding the well-established mTOR signaling pathway and its ever-expanding cellular roles.

The clinical impact of mTOR inhibitors has been profound, contributing to the treatment of a wide range of human diseases, including cancers, tuberous sclerosis complex (TSC), and Alzheimer’s disease [29]. Numerous studies have implicated mTOR’s catalytic kinase domain in drug development. Specifically, inhibitors against the kinase domain of mTOR have been developed as second-generation mTOR inhibitors. These inhibitors have shown significant antitumor activities both in vivo and in vitro, making mTOR a promising target to develop more effective therapeutic strategies [30,31]. The binding of ATP to the mTOR kinase domain is particularly important for mTOR’s cellular functions, such as growth and metabolism [32]. These active site inhibitors effectively attenuate mTOR activity by concurrently targeting both mTOR signaling and its associated feedback loops, ensuring complete inhibition of the mTOR signaling pathway and, consequently, mitigating disease progression [33,34,35,36,37]. Sapanisertib, an example of a potent dual inhibitor of mTORC1 and mTORC2 signaling, has been shown to possess antitumor roles in renal cell carcinoma, downregulating the expression levels of mTOR’s downstream targets [38,39,40,41,42]. Others have demonstrated the use of sapanisertib in clinical trials in conjugation with metformin, a widely used drug for treating T2D that also exhibits mTOR inhibitory functions, which could further enhance the antitumor effects, preventing cancer progression [43,44,45,46]. Other ATP-competitive mTOR inhibitors have also been identified, such as pyrazolopyrimidines (e.g., PP242 and PP30) and KU0063794, which have demonstrated promising results in suppressing cell cycle progression and halting cell proliferation [47].

In the present study, we screened the Traditional Chinese Medicine (TCM) database (http://tcm.cmu.edu.tw/, accessed on 21 April 2024) to identify potential ATP-competitive agonists that target the mTOR-kinase domain (mTOR-KD) [48,49,50,51]. The TCM database enlists natural products with medicinal properties that capture the relationships between drugs, targets, and diseases. Furthermore, the conformational stability and dynamic features of the mTOR-KD bound to the TCM-selected compounds were tested by subjecting each complex to 200 ns molecular dynamic (MD) simulations and molecular mechanics/generalized Born surface area (MM/GBSA) to extract free binding energies. This study aims to demonstrate the effectiveness of two selected natural products that could compete with ATP to bind the mTOR-KD and serve as potential potent mTOR inhibitors. Such inhibitors could serve as possible therapeutic targets for regulating mTOR function and mitigating human disease progression.

## 2. Results and Discussion

### 2.1. mTOR Kinase Domain Structural Modeling

**Figure 1:**
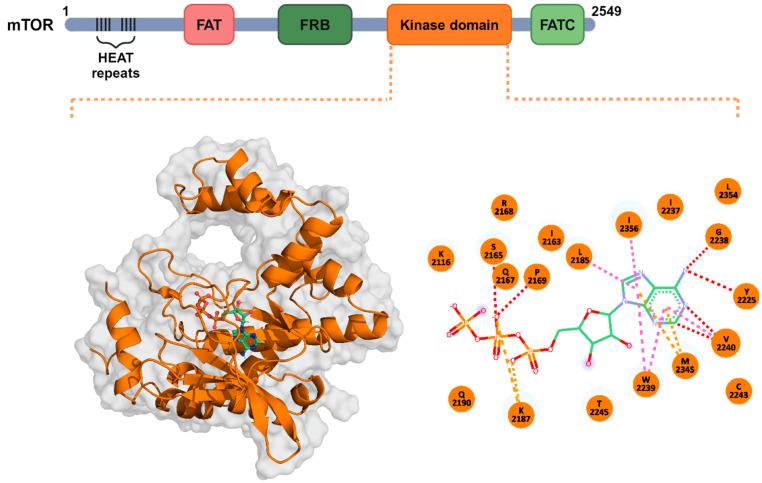
The structural organization of mTOR and its ATP binding sites. A schematic representation of the four mTOR domains; FAT, FRB, kinase domain (KD), and FATC. The 3D structure of the KD is depicted in orange, bound to ATP (green). The 2D schematic of ATP interatomic interactions with the KD residues formed H-bonds (red), electrostatic interactions (magenta), and pi-bonds (orange). mTOR, mammalian target of rapamycin; HEAT, huntingtin, elongation factor 3, PP2A, and TOR1; FAT, FRAP, ATM, and TRAP; FRB, FKBP–rapamycin binding; KD, kinase domain; FATC, C-terminal FAT.

The mTOR structure comprises multiple domains, which include a huntingtin, elongation factor 3, a subunit of protein phosphatase 2A, TOR1 (HEAT) domain, a FRAP, ATM, TRRAP (FAT) domain, a FKBP–rapamycin binding (FRB) domain, a kinase domain (KD), and a C-terminal FAT (FATC) domain (Figure 1). The ATP-binding KD is ~280 residues long and is essential for overall mTOR functions [52]. As such, in recent years, the ATP-binding KD has become an attractive drug target for inhibiting mTOR function. In our study, we extracted the mTOR-KD from the cryo-EM structure of (PDB ID: 6ZWM) (Figure 1) to identify compounds from the TCM natural product databases that can disrupt the ATP interaction with the mTOR-KD.

**Figure 2:**
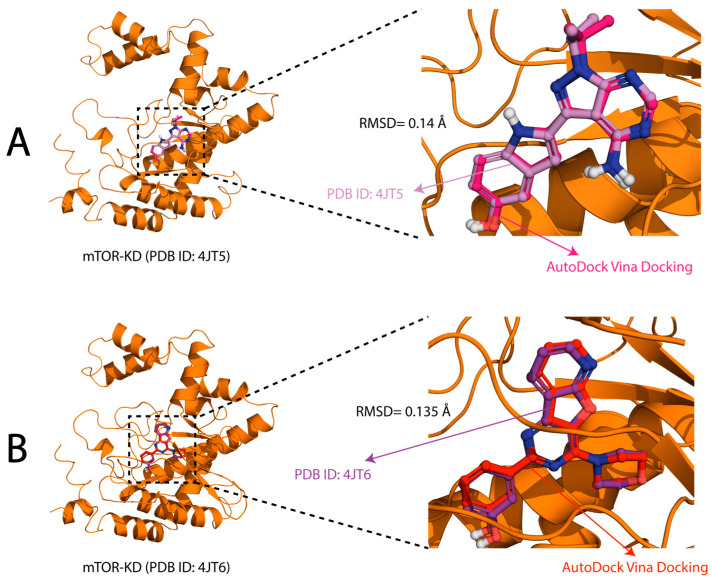
Validation of the protocol using experimental ligands. (**A**) The superimposed binding of 2-[4-amino-1-(propan-2-yl)-1H-pyrazolo[3,4-d]pyrimidin-3-yl]-1H-indol-5-ol (PDB ID: 4JT5) (lavender) and the docked ligand (magenta); (**B**) the binding of 3-(4-morpholin-4-ylpyrido[3′,2′:4,5]furo[3,2-d]pyrimidin-2-yl)phenol (PDB: ID 4JT6) (purple) and the docked ligand (red).

To validate the docking protocol before screening for compounds from the TCM natural product database, we examined the structure of the mTOR-KD domain with co-crystallized ligands 2-[4-amino-1-(propan-2-yl)-1H-pyrazolo [3,4-d]pyrimidin-3-yl]-1H-indol-5-ol (PDB ID: 4JT5) and 3-(4-morpholin-4-ylpyrido[3′,2′:4,5]furo[3,2-d]pyrimidin-2-yl)phenol (PDB: ID 4JT6) (Figure 2A,B) [52]. As presented in Figure 2, the superimposed ligands perfectly align with the experimental conformation. The figures show that the experimental and docking predictions overlaid with a high degree of alignment, which determines the accuracy and reliability of the docking protocol in reproducing experimental binding poses. Furthermore, the RMSD between the co-crystallized ligands and the docked ligand for PDB structures 4JT5 and 4JT6 were 0.14 and 0.135 Å, respectively, confirming that AutoDock Vina can accurately predict ligand–protein interactions, supporting its use in this study.

### 2.2. Discovery of the mTOR-KD Inhibitors by Screening the TCM Library

The mTOR KD-ATP binding site was targeted with compounds from the TCM library via a multi-step computational screening approach using AutoDock Vina scoring function in SMINA tools. The TCM compound library has previously been used as a source to treat both communicable and non-communicable diseases [53]. The compounds were retrieved from the database and underwent absorption, distribution, metabolism, excretion, and toxicity (ADMET) analysis, whereby 35,474 compounds obeyed the Lipinski rule of five. Furthermore, with the docking scores set to a threshold of ≥−5 kcal/mol, 19,541 presented compounds fit the selection criteria. To further narrow the compound selection, the docking threshold was increased in the second round of screening to ≥−14 kcal/mol, resulting in 45 compounds. These remaining compounds were subjected to induced-fit docking (IFD), with four compounds showing the highest docking scores selected to inhibit ATP binding to the mTOR-KD (Table 1). Two compounds presented the highest docking scores, out of which 1-hydroxy-4-(3-hydroxypropyl)-2-[(2R,3S,4R,5R,6S)-3,4,5-trihydroxy-6 (hydroxymethyl)oxan-2-yl] oxyanthracene-9,10-dione (which we refer to as Compound A throughout the manuscript) and 2-[3,4-dihydroxy-5-[(2R,3S,4R,5R,6S)-3,4,5-trihydroxy-6-(hydroxymethyl)oxan-2-yl]oxyphenyl]-5,7-dihydroxychromen-4-one (which we refer to as Compound B throughout the manuscript) in complex with the mTOR-KD underwent MD simulations to measure their conformational dynamics and stability.

### 2.3. Binding Modes of the Selected TCM Compounds to the mTOR-KD

#### 2.3.1. Compound A

**Figure 3:**
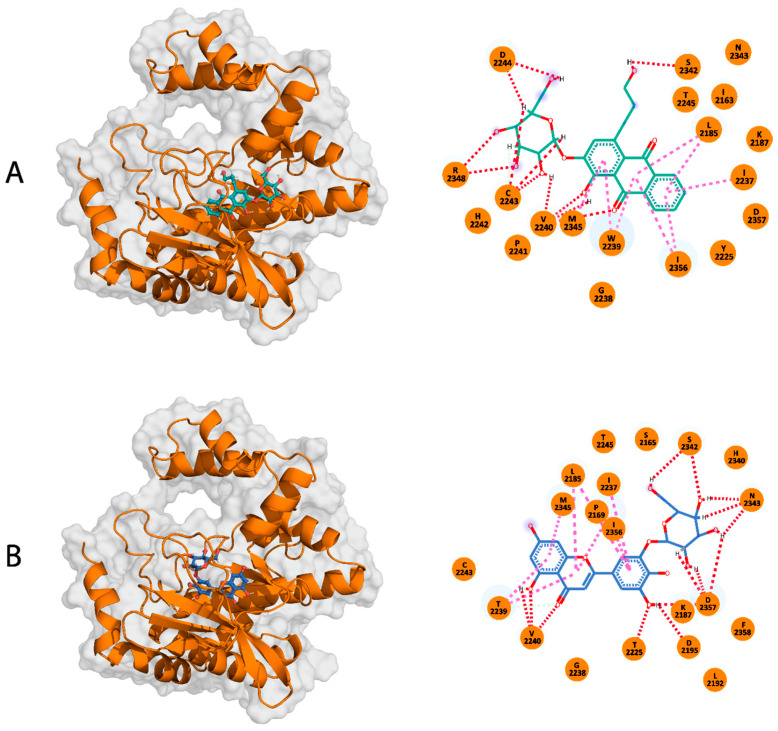
The interaction patterns of Compounds A and B with the mTOR-KD. (**A**) The right panel shows a 3D representation of the mTOR-KD (orange) bound to Compound A (teal), and the left panel depicts a 2D representation of the Compound A-mTOR-KD interaction. (**B**) The right panel shows a 3D representation of the mTOR-KD (orange) bound to Compound B (blue), and the left panel depicts a 2D representation of the Compound B-mTOR-KD interaction. The H-bonds are represented by red dashed lines, and magenta dashed lines indicate electrostatic interactions.

Compound A is an organic compound identified as an anthraquinone glycoside found in Rubiaceae, Rhamnaceae, and Polygonaceae families of plants [54,55]. Anthraquinone glycoside biosynthesis occurs via the polyketide pathway, followed by glycosylation [54]. Anthraquinone glycoside compounds tend to possess medicinal features such as anti-inflammatory and antimicrobial effects [55], implicating their benefit in treating infectious diseases [56] and various inflammatory conditions [57] (Table 2). Compound A also has a tricyclic aromatic molecule with carbonyl groups, known as an anthraquinone core with a glycoside (2R,3S,4R,5R,6S)-3,4,5-trihydroxy-6-(hydroxymethyl)oxan-2-yl group. The anthraquinone core is attached to a 3-hydroxypropyl group and one hydroxyl group. With a docking score of −69.2 kcal/mol, different parts of Compound A formed 11 H-bonds with five mTOR-KD residues (V2240, D2244, C2243, S2342, and R2348). In addition, 10 ionic interactions were formed with residues L2185, I2237, W2239, L2345, and I2356 (Figure 3A). Similar to ATP, Compound A formed interactions with six residues, which include L2185, I2237, W2239, V2240, C2243, and I2356, indicating that Compound A and ATP possess a comparable interaction pattern to the mTOR-KD.

From a pharmacological perspective, the structure of Compound A, in particular the oxan-2-yl, could improve its pharmacokinetics and water solubility, enhancing its bioavailability [58]. The 3-hydroxypropyl side chain can modify the lipophilic properties of the compound and its ability to interact with specific protein targets or cellular membranes [56]. Nonetheless, Compound A’s biological significance comes from its 9,10-dione core structure, which gives it potential antimicrobial and anti-cancer properties [55]. Furthermore, having both multiple hydroxyl groups and possessing free radical scavenging properties could affect its ability to interact with other biological targets and serve as a potential antioxidant agent, respectively [59].

#### 2.3.2. Compound B

Compound B is a flavonoid derivative organic compound composed of a chromen-4-one flavonoid core [60]. Flavonoid compounds have been reported to exhibit antioxidant, anti-inflammatory, and other health-beneficial properties [61] (Table 2). Compound B has structural similarities with other known flavonoid glycosides commonly found in plants, such as quercetin derivatives [62,63]. The core of this molecule is formed by two aromatic rings linked via a heterocyclic ring with an oxygen atom. Additionally, it has several hydroxyl groups, with two attached to the chromen-4-one structure and two connected to the phenyl rings. As a flavonoid glycoside, its structure has functional implications whereby the glycoside core enhances its solubility and bioavailability, and the hydroxyl groups indicate its antioxidant activity [60,61]. Compound B displayed a docking score of −69.2 kcal/mol, with residues K2187, D2195, Y2225, V2240, S2342, N2343, and D2357 forming H-bonds with the mTOR-KD. Meanwhile, residues P2169, I2237, W2239, M2345, I2356, and L2815 formed ionic interactions (Figure 3B). Similar to both ATP and Compound A, Compound B interacted with the mTOR-KD through residues P2169, L2185, K2187, I2237, W2239, Y2225, V2240, L2354, and I2356. Such interactions emphasize the conserved residues between ATP, Compound A, Compound B, and the kinase domain of mTOR.

### 2.4. Dynamic Stability and Compactness Assessment of the mTOR-KD Ligand Binding

The conformational stability and dynamics of the mTOR-KD bound to Compounds A and B were elucidated by running 200 ns MD simulations. The root mean square deviation (RMSD) trajectories of the Cα-atoms demonstrate the degree to which the protein conformation has changed over time. The radius of gyration (Rg) trajectories illustrates the degree of compactness of the mTOR-KD bound to Compounds A and B, revealing essential information regarding the binding and unbinding events during the MD simulation.

**Figure 4:**
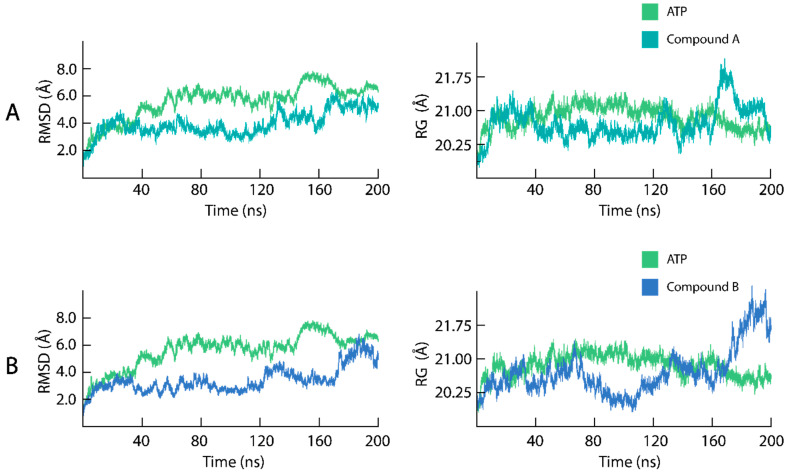
Dynamic stability and compactness assessment of ligands bound to the mTOR-KD. (**A**) RMSD and Rg of Compound A (teal) in complex with the mTOR-KD. (**B**) RMSD and Rg of Compound B (blue) in complex with the mTOR-KD. The dynamic stability of Compounds A and B were compared with the RMSD and Rg of ATP in complex the mTOR-KD (green).

Primarily, the RMSD and Rg trajectories of the mTOR-KD in complex with endogenous ATP were measured to compare the stability of the structure with the mTOR bound to Compounds A and B (Figure 4A,B). Initially, the RMSD of the mTOR-KD bound to ATP increased from 1.8 to 4.0 Å in the first 20 ns of the simulation, after which the system fluctuated from 20 to 40 ns, with the RMSD reaching 5.8 Å. From 40 to 60 ns, the mTOR-KD–ATP complex fluctuated, reaching 6.2 Å, and, subsequently, from 60 to 150 ns, the complex stabilized with an RMSD between 6.0 and 6.2 Å, with a minor convergence at 70 ns. From 150 to 170 ns, the mTOR-KD–ATP complex showed a significant convergence in the system, with the RMSD reaching 8.0 Å, followed by a return to equilibrium at an RMSD of 6.0 Å until the end of the 200 ns simulation. In addition, the Rg measurements of the mTOR-KD–ATP demonstrated the compactness of the complex during the 200 ns simulation, with the Rg values averaging between 20.25 and 21.0 Å. The Rg fluctuated during the initial 40 ns of the simulation, stabilizing at 21.0 Å from 40 to 160 ns, with a decrease in Rg at 140 ns to 20.25 Å. For the remainder of the simulation, the Rg values decreased to 20.35 Å, indicative of a compact mTOR-KD–ATP complex. The structural compactness corroborated with the RMSD results, demonstrating that the mTOR-KD structure bound to the endogenous ATP formed a stable compact system.

The mTOR-KD in complexes with Compounds A and B demonstrated stable structures (Figure 4A,B). Initially, the mTOR-KD–Compound A complex RMSD increased from 1.8 to 5.0 Å in the first 30 ns of the MD simulation. Subsequently, from 35 to 130 ns of the simulation, the RMSD decreased to 3.8 ns, where the mTOR-KD–Compound A complex stabilized between 3.8 and 4.0 Å. From 130 to 160 ns, the complex fluctuated between 3.8 and 5.0 Å, after which the RMSD increased to 5.8 Å at 170 ns. In the final stages of the simulation, the RMSD stabilized at 5.0 Å for the mTOR-KD–Compound A complex, which, on average, is lower than that of the mTOR-KD–ATP. Similarly, the mTOR-KD–Compound B formed a more stable complex than that of mTOR–ATP (Figure 4B). The RMSD increased from 1.8 to 3.0 Å in the first 35 ns of the simulation, from which the RMSD decreased to 2.0 Å at 40 ns. From 40 to 80 ns, the mTOR-KD–Compound B RMSD fluctuated between 2.0 and 4.0 Å, whereas, from 80 to 120 ns, the complex stabilized with a constant RMSD of 3.0 Å. Next, the mTOR-KD–Compound B complex converged from 120 to 130 ns with an increase in RMSD to 4 Å, then decreased to 2.5 Å at 170 ns. In the final 30 ns of the 200 ns simulation, the system converged to 6 Å at 185 ns, decreasing back to 4.0 Å at 200 ns.

The measurement of the Rg to elucidate the structural compactness of the mTOR-KD in complexes with Compounds A and B is displayed over the 200 ns simulation (Figure 4A,B). The Rg of the mTOR-KD–Compound A complex (Figure 4A) fluctuated during the initial 40 ns of the simulation, with an increase in Rg from 20.0 to 21.0 Å in the initial 5 ns. From 5 to 40 ns, the Rg fluctuated between 20.5 and 21.5 Å, after which the complex stabilized from 40 to 120 ns, with an average Rg of 20.25 Å. Subsequently, from 120 ns until the end of the simulation, the mTOR-KD–Compound A complex experienced a significant fluctuation, with Rg reaching 28.0 Å, before the complex stabilized with an Rg of 20.35 Å at 200 ns. The mTOR-KD complex with Compound A demonstrated moderate fluctuations in the Rg values compared to ATP, which may indicate that mTOR, when bound to Compound A, causes higher conformational flexibility than the mTOR-KD–ATP complex. As for the mTOR-KD–Compound B complex, high fluctuations in Rg were observed throughout the 200 ns simulation (Figure 4B). In the initial 70 ns of the simulation, the Rg values of the mTOR-KD–Compound B complex displayed a similar profile to the mTOR-KD–ATP complex, increasing from 20.25 and 21.0 Å. However, after 70 ns, the mTOR-KD–Compound B complex demonstrated significant deviations in the Rg, whereby it decreased to 19.5 Å at 110 ns and then increased to 22.5 ns at the end of the 200 ns simulation. The fluctuations in Rg observed for the mTOR-KD–Compound B complex suggest that the system experiences significant changes in structural compactness, indicating that Compound B could cause more dynamic structural rearrangements or instability in the mTOR-KD structure compared to ATP and Compound A.

The RMSD analysis for the mTOR-KD bound to Compounds A and B demonstrated stable complexes compared to those of the mTOR-KD–ATP complex (Figure 4A,B). In contrast, the Rg analysis of the mTOR-KD–Compounds A and B complexes demonstrated fluctuations during the 200 ns MD simulation. In general, if the RMSD of the protein–ligand complex is high, some protein regions are expanding, mainly the unstructured loop regions [64]. In addition, ligand binding fluctuations in Rg, despite having a stable RMSD, could indicate that the protein has the potential for induced fit upon ligand binding [65]. This flexibility allows the protein to adapt its shape to accommodate ligands [66].

### 2.5. Residue Flexibility Analysis of the mTOR-KD Ligand Binding

**Figure 5:**
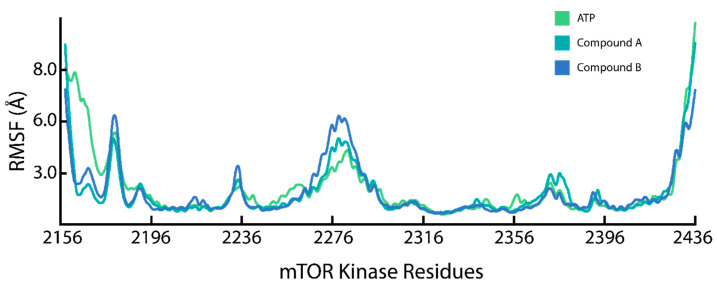
Residue flexibility assessment of the mTOR-KD ligand complexes. Residual flexibility of the mTOR-KD in complex with ATP (green), Compound A (teal), and Compound B (blue).

The root mean square fluctuations (RMSF) of the Cα-atoms of the mTOR-KD in complex with ATP, Compound A, and Compound B measure the flexibility and conformational deviation of the residues from their mean position throughout a 200 ns simulation (Figure 5). Subsequently, RMSF calculates the dynamic activities of specific regions, whereby it can establish the optimized conformational states of the mTOR-KD bound to ligands. The mTOR-KD N-terminal region between residues 2156 and 2196 demonstrated significant fluctuations across all three compounds, with ATP showing the highest RMSF. The N-terminal region of the mTOR-KD is necessary for ATP binding and catalytic activity [52], where we can observe the binding of the ATP phosphate group to residues in this region (Figure 1). In addition, the RMSF fluctuated between residues 2226 and 2240 for both the mTOR-KD Compound A and B complexes, which can result from the interactions with residues I2237 and T2239. Between residues 2236 and 2276, there is a noticeable increase in RMSF, especially with Compound B, suggesting enhanced flexibility or movement. This flexibility could be related to the catalytic cleft, where substrate processing occurs. The variability between compounds indicates different binding modes or induced conformational changes [67], whereas, between residues 2356 and 2436, the RMSF increases, particularly with ATP and Compound B. This flexibility could result from the flexible loop region of the mTOR-KD being less structured and more exposed to solvent interactions. The regions of the mTOR-KD that displayed the highest fluctuations reflect the ligand–protein binding sites [52].

### 2.6. Binding Free Energy Estimation of the mTOR-KD with Ligands

To substantiate the RMSD, Rg, and RMSF data of mTOR-KD binding to ATP and Compounds A and B, we applied the MM/GBSA method to calculate the total binding free energy (ΔG) to DNA. The MM/GBSA method gives a more robust and accurate account of binding energies than the conventional docking scores. As such, the MM/GBSA estimates the binding free energy of Compounds A and B in complex with the mTOR-KD (Table 3) compared to the mTOR-KD–ATP complex.

The MM/GBSA results (Table 3) have demonstrated that both Compounds A and B in complex with the mTOR-KD show stronger van der Waals interactions Vdw (−41.15 and −40.92 kcal/mol) compared to the mTOR-KD–ATP (−30.83 kcal/mol). This suggests that Compounds A and B form more favorable non-polar contacts with the mTOR-KD binding site. The electrostatic interactions (EEL) showed that Compound B has the most favorable EEL (−99.52 kcal/mol) in comparison to ATP (−81.28 kcal/mol) and Compound A (−40.45 kcal/mol), which indicates that Compound B has stronger charge–charge or dipole interactions with the mTOR-KD. Furthermore, ATP and Compound B presented the highest polar solvation energy (104.94 and 91.88 kcal/mol) compared to Compound A (49.28 kcal/mol), suggesting that ATP experiences more unfavorable desolvation upon binding to the mTOR-KD. With the non-polar solvation energy (ESURF), Compound B presented the most favorable non-polar solvation with the mTOR-KD (−7.67 kcal/mol), which correlates with its strong Vdw interactions. As for the ΔG gas, Compound B presented the most favorable ΔG binding (−140.45 kcal/mol), resulting from the strong EEL between Compound B and the mTOR-KD. In contrast, the ΔG solvent showed that ATP has the highest solvation penalty (100.55 kcal/mol), while Compound A has the lowest (43.25 kcal/mol), consistent with their ESURF. Meanwhile, total binding free energy (ΔG) provides a thermodynamic evaluation of the strength and stability of the above-mentioned molecular interaction, whereby Compound B showed the most favorable ΔG (−56.23 kcal/mol), followed by Compound A (−38.36 kcal/mol), and ATP (−11.56 kcal/mol), signifying that both TCM Compounds A and B bind more robustly to the mTOR-KD than the endogenous molecule ATP [68,69,70].

## 3. Materials and Methods

### 3.1. Structures, Sequence Retrieval, and Modeling

The Protein Databank (http://www.rcsb.org/, accessed on 10 April 2024) was used to retrieve the X-ray crystal structure of the mTOR-KD using PDB ID: 6ZWM. The mTOR-KD structure was prepared and minimized using Chimera and AMBER simulation packages using the FF14SB force field. To screen the potential ligand against the mTOR-KD, we utilized compounds from the TCM library (http://tcm.cmu.edu.tw/, accessed on 10 April 2024) [48,49,50,51].

### 3.2. Structures and Validation of Molecular Screening

Before structural ligand screening for compounds targeting the mTOR-KD domain, we validated our docking method using X-ray crystal structure of the mTOR-KD bound to compounds 2-[4-amino-1-(propan-2-yl)-1H-pyrazolo[3,4-d]pyrimidin-3-yl]-1H-indol-5-ol (PDB ID: 4JT5) and 3-(4-morpholin-4-ylpyrido[3′,2′:4,5]furo[3,2-d]pyrimidin-2-yl)phenol (PDB: ID 4JT6) [52]. The compounds were stripped from the mTOR-KD X-ray structure and docked again using the Autodock Vina scoring function in SMINA. To compare the docking positions of the X-ray crystal structures of the mTOR-bound compounds and the AutoDock-Vina-bound compounds, we used the PyMOL 3D visualization tool. To validate the docking protocol, superimposition of the experimental and docked conformation was performed for each ligand, and the differences were calculated.

### 3.3. Molecular Screening of Natural Product Libraries

The TCM ligand library was downloaded and processed to generate the correct required format for target screening [48,49,50,51]. Initially, the database was screened for the Lipinski rule of five (R5) to filter out the violating and toxic compounds using the FAF4 drug online webserver. After this, the obtained R5-obeying compounds from the TCM database were screened against the active site of the mTOR-KD using the AutoDock Vina scoring function (SMINA) [71]. The most favorable hits to the mTOR-KD from SMINA screening were further screened using the AutoDock Vina scoring function (ADFR), which performed flexible docking of the compounds with increased accuracy [72]. Finally, the top three hits from the TCM database were subjected to visual analysis using PyMOL and Discovery Studio visualizer (free academic version for visualization) and MD simulation for further validation [73,74].

### 3.4. Molecular Simulation of Top Scoring Hits

The ligands from the TCM database with the highest docking scores with the mTOR-KD were subjected to MD simulations and MM/GBSA free-energy-calculation-based validation using AMBER20 [75,76]. The drug topologies were generated and processed for simulation using antechamber and parmchk2 [77]. The topology and structural coordinates files were used to minimize each complex in two stages, as follows: (1) minimization of 12,000 steps; and (2) minimization of 6000 steps. Each complex was sequentially heated and equilibrated for 50 ns. In the production stage, a 200 ns simulation for each complex was performed, the simulation was accelerated by using the GPU version of PMEMD.cuda, and trajectories were processed using PTRAJ and CPPTRAJ [78,79].

### 3.5. Post-Simulation Analysis of the Protein–Ligand Complexes

The stability of the ligand in the mTOR-KD complex was calculated as the root mean square deviation (RMSD), whereas the flexibility index of each residue was measured through root mean square flexibility (RMSF). In addition, the mTOR-KD–ligand complex size was calculated as the radius of gyration (Rg).

(1) $$RMSD=\sqrt{\frac{1}{N}\;\sum_{i=1}^{N}\delta_{i}^{2}}$$

The value of δi represents the distance between atom *i* and either a reference structure or the average position of N equivalent atoms. This parameter is computed for protein backbone heavy atoms, N, O, and Cα, or occasionally only for the Cα atoms.

(2) $$r_{RG}^{2}=\frac{\sum_{i=1}^{N}m_{i}{\left(r_{i}-r_{CM}\right)}^{2}}{\sum_{i=1}^{N}m_{i}}$$

where:

- **r**_i_ is the position of the atom at index *i;*
- *m*_i_ is the mass of the atom at index *i;*
- **r**_CM_ is the center of mass;
- N is the number of atoms being counted;
- r^2^_RG_ is the square of the radius of gyration.

For residue flexibility analysis, the following mathematical expression was used:

(3) $$B=\frac{{8\pi }^{2}}{3}\left\langle ∆r^{2}\right\rangle$$

where

- B is B-factor, and⟨Δr^2^⟩ is the mean square deviation (i.e., ⟨Δr^2^⟩ = RMSD^2^).
- By rearranging the above equation and accounting for 3 spatial dimensions, we can obtain the RMSF as follows:

(4) $$RMSF=\sqrt{\frac{3B}{{8\pi }^{2}}}$$

### 3.6. Binding Free Energy Calculation Using the MM/GBSA Approach

The end-point total binding free energy was estimated using the molecular mechanics/generalized Born surface area (MM/GBSA) method, which is arguably the most extensively employed and accurate approach. Using the MMPBSA.py script, the stabilized fractions from the simulation trajectories were subjected to free energy calculations [80]. The binding energies were calculated as the difference between the free energies complex (*G_complex_, _solvated_*) and the unbound states of the receptor (*G_mTOR_*_, *solvated*_) and ligand (*G_ligand, solvated_*). The following equation calculates each term in the total binding energy:

(5) $${∆G}_{bind}=G_{\left(complex,\;\;\;solvated\right)}-G_{\left(mTOR,\;\;\;solvated\right)}-G_{\left(ligand\;\;\;solvated\right)}$$

## 4. Conclusions

The present study identifies two natural products, Compounds A and B, as competitive ATP agonists to mTOR’s catalytic KD. The interatomic interactions of Compounds A and B to the mTOR-KD were similar to ATP–mTOR interactions. Furthermore, we have demonstrated the effectiveness of both compounds in forming stable complexes with the mTOR-KD, which is more effective than the mTOR-KD–ATP complex, with more robust binding affinities. Compound B, which presented tighter binding than both ATP and Compound A, is part of the flavonoid family, which has been shown to possess anti-cancer properties. Such compounds could serve as potential mTOR inhibitors, acting as therapeutic agents in regulating mTOR overall functions. Recent mTOR inhibitors that target multiple domains of both mTORC1 and mTORC2 complexes have emerged. For instance, RapaLink-1 resembles rapamycin in structure has been shown to be a potent mTORC1 inhibitor. Wang et al. demonstrated the therapeutic efficacy of RapaLink-1 in organ transplantation, where it was superior to rapamycin’s inhibitory role in prolonging graft survival and displayed a reduced graft rejection rate [81]. Like the studies conducted using RapaLink-1, we believe that it is important to translate the in silico work presented in this manuscript to experiential and clinical studies in order to investigate the potential roles of both Compounds A and B in regulating both mTORC1 and mTORC2 signaling. Investigating the different classes of mTOR inhibitors and understanding the roles of such agonists is crucial for developing therapeutic strategies against human diseases and mitigating their progression.

## Figures and Tables

**Table 1:** Top hits identified through muti-step screening and rescoring via the IFD method. The table presents the 2D structures, compound names, and docking scores of the top two hits.

2D Structure	Compound Name	Docking Scores	Identifier
** 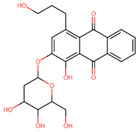 **	1-hydroxy-4-(3-hydroxypropyl)-2-[(2R,3S,4R,5R,6S)-3,4,5-trihydroxy-6-(hydroxymethyl)oxan-2-yl]oxyanthracene-9,10-dione	−15.455	A
** 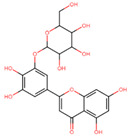 **	2-[3,4-dihydroxy-5-[(2R,3S,4R,5R,6S)-3,4,5-trihydroxy-6-(hydroxymethyl)oxan-2-yl]oxyphenyl]-5,7-dihydroxychromen-4-one	−14.886	B

**Table 2:** ADMET analysis results with their biological and toxicological properties.

Molecular. Formula	Compound	MW. (g/mol)	Source	Molecule Class	Biological Activity	Lipinski Violation	Pfizer Rule *	** AMES Toxicity	*** IGC_50_	HBD	HBA	Rotatable Bonds No.	TPSA (Å^2^)	Bioactivity
C_23_H_24_O_10_	Compound A	460.44	Rubiaceae, Rhamnaceae, and Polygonaceae families	Anthraquinone	Antimicrobial	1	pass	++	4.638	6	10	6	174	0.45
C_21_H_20_O_12_	Compound B	464.38	*Ocimum sanctum, Phyllostachys* species	Flavonoid	Anti-inflammatory	2	pass	+	3.878	8	12	4	211	0.44

* Compounds with logP > 3 and TPSA < 75 are likely to be toxic. ** The output value is the probability of being toxic. *** Tetrahymena pyriformis 50% growth inhibition concentration defined as −log10 [(mg/L)/(1000 × MW)].

**Table 3:** Binding free energy calculated as MM/GBSA of ATP, Compound A, and Compound B with SD. All values are presented in kcal/mol.

Parameters	ATP	Compound A	Compound B
VDWAALS	−30.83 ± 0.20	−41.15 ± 0.13	−40.92 ± 0.18
EEL	−81.28 ± 1.25	−40.45 ± 0.34	−99.52 ± 0.62
EGB	104.94 ± 1.12	49.28 ± 0.27	91.88 ± 0.37
ESURF	−4.39 ± 0.03	−6.03 ± 0.01	−7.67 ± 0.01
DELTA G gas	−112.11 ± 1.27	−81.61 ± 0.36	−140.45 ± 0.57
DELTA G solv	100.55 ± 1.12	43.25 ± 0.26	84.21 ± 0.36
DELTA TOTAL	−11.56 ± 0.46	−38.36 ± 0.18	−56.23 ± 0.31

## Data Availability

The datasets included in this study are not available for sharing from the corresponding authors due to unpublished data and ethical restrictions by the institute.

## References

[1] Leung A., Rangamani P. (2023). Computational modeling of AMPK and mTOR crosstalk in glutamatergic synapse calcium signaling. NPJ Syst. Biol. Appl..

[2] Tee A.R., Blenis J., Proud C.G. (2005). Analysis of mTOR signaling by the small G-proteins, Rheb and RhebL1. FEBS Lett..

[3] Populo H., Lopes J.M., Soares P. (2012). The mTOR signalling pathway in human cancer. Int. J. Mol. Sci..

[4] Marques-Ramos A., Cervantes R. (2023). Expression of mTOR in normal and pathological conditions. Mol. Cancer.

[5] Saxton R.A., Sabatini D.M. (2017). mTOR Signaling in Growth, Metabolism, and Disease. Cell.

[6] Zoncu R., Efeyan A., Sabatini D.M. (2011). mTOR: From growth signal integration to cancer, diabetes and ageing. Nat. Rev. Mol. Cell Biol..

[7] Zhou Y., Zhou Z., Peng J., Loor J.J. (2018). Methionine and valine activate the mammalian target of rapamycin complex 1 pathway through heterodimeric amino acid taste receptor (TAS1R1/TAS1R3) and intracellular Ca(2+) in bovine mammary epithelial cells. J. Dairy Sci..

[8] Pende M., Kozma S.C., Jaquet M., Oorschot V., Burcelin R., Le Marchand-Brustel Y., Klumperman J., Thorens B. (2000). Hypoinsulinaemia, glucose intolerance and diminished beta-cell size in S6K1-deficient mice. Nature.

[9] Xin-Long C., Zhao-Fan X., Dao-Feng B., Wei D. (2011). mTOR partly mediates insulin resistance by phosphorylation of insulin receptor substrate-1 on serine(307) residues after burn. Burns.

[10] Yu W., Li C., Zhang D., Li Z., Xia P., Liu X., Cai X., Yang P., Ling J., Zhang J. (2022). Advances in T Cells Based on Inflammation in Metabolic Diseases. Cells.

[11] Hara K., Maruki Y., Long X., Yoshino K.-I., Oshiro N., Hidayat S., Tokunaga C., Avruch J., Yonezawa K. (2002). Raptor, a binding partner of target of rapamycin (TOR), mediates TOR action. Cell.

[12] Kim D.H., Sarbassov D.D., Ali S.M., King J.E., Latek R.R., Erdjument-Bromage H., Tempst P., Sabatini D.M. (2002). MTOR interacts with Raptor to form a nutrient-sensitive complex that signals to the cell growth machinery. Cell.

[13] Loewith R., Jacinto E., Wullschleger S., Lorberg A., Crespo J.L., Bonenfant D., Oppliger W., Jenoe P., Hall M.N. (2002). Two TOR complexes, only one of which is rapamycin sensitive, have distinct roles in cell growth control. Mol. Cell.

[14] Jacinto E., Loewith R., Schmidt A., Lin S., Rüegg M.A., Hall A., Hall M.N. (2004). Mammalian TOR complex 2 controls the actin cytoskeleton and is rapamycin insensitive. Nat. Cell Biol..

[15] Kaizuka T., Hara T., Oshiro N., Kikkawa U., Yonezawa K., Takehana K., Iemura S.-I., Natsume T., Mizushima N. (2010). Tti1 and Tel2 are critical factors in mammalian target of rapamycin complex assembly. J. Biol. Chem..

[16] Ma X.J.M., Blenis J. (2009). Molecular mechanisms of mTOR-mediated translational control. Nat. Rev. Mol. Cell Biol..

[17] Oh W., Wu C.-C., Kim S.J., Facchinetti V., Julien L.-A., Finlan M., Roux P., Su B., Jacinto E. (2010). mTORC2 can associate with ribosomes to promote cotranslational phosphorylation and stability of nascent Akt polypeptide. EMBO J..

[18] Deng Y.F., Wu S.T., Peng H.Y., Tian L., Li Y.N., Yang Y., Meng M., Huang L.-L., Xiong P.-W., Li S.-Y. (2023). mTORC2 acts as a gatekeeper for mTORC1 deficiency-mediated impairments in ILC3 development. Acta Pharmacol. Sin..

[19] Dibble C.C., Asara J.M., Manning B.D. (2009). Characterization of Rictor Phosphorylation Sites Reveals Direct Regulation of mTOR Complex 2 by S6K1. Mol. Cell. Biol..

[20] Tzatsos A., Kandror K.V. (2006). Nutrients suppress phosphatidylinositol 3-kinase/Akt signaling via raptor-dependent mTOR-mediated insulin receptor substrate 1 phosphorylation. Mol. Cell. Biol..

[21] Marafie S.K., Al-Shawaf E.M., Abubaker J., Arefanian H. (2019). Palmitic acid-induced lipotoxicity promotes a novel interplay between Akt-mTOR, IRS-1, and FFAR1 signaling in pancreatic β-cells. Biol. Res..

[22] Sarbassov D., Ali S.M., Sengupta S., Sheen J.-H., Hsu P.P., Bagley A.F., Markhard A.L., Sabatini D.M. (2006). Prolonged rapamycin treatment inhibits mTORC2 assembly and Akt/PKB. Mol. Cell..

[23] Chang I., Loo Y.-L., Patel J., Nguyen J.T., Kim J.K., Krebsbach P.H. (2024). Targeting of lysosomal-bound protein mEAK-7 for cancer therapy. Front. Oncol..

[24] Nguyen J.T., Haidar F.S., Fox A.L., Ray C., Mendonça D.B., Kim J.K., Krebsbach P.H. (2019). mEAK-7 Forms an Alternative mTOR Complex with DNA-PKcs in Human Cancer. iScience.

[25] Nguyen J.T., Ray C., Fox A.L., Mendonça D.B., Kim J.K., Krebsbach P.H. (2018). Mammalian EAK-7 activates alternative mTOR signaling to regulate cell proliferation and migration. Sci. Adv..

[26] Mendonca D.B., Nguyen J.T., Haidar F., Fox A.L., Ray C., Amatullah H., Liu F., Kim J.K., Krebsbach P.H. (2020). MicroRNA-1911-3p targets mEAK-7 to suppress mTOR signaling in human lung cancer cells. Heliyon.

[27] Smithson L.J., Gutmann D.H. (2016). Proteomic analysis reveals GIT1 as a novel mTOR complex component critical for mediating astrocyte survival. Genes Dev..

[28] Harwood F.C., Geltink R.I.K., O’hara B.P., Cardone M., Janke L., Finkelstein D., Entin I., Paul L., Houghton P.J., Grosveld G.C. (2018). ETV7 is an essential component of a rapamycin-insensitive mTOR complex in cancer. Sci. Adv..

[29] Karki R., Man S.M., Malireddi R.S., Kesavardhana S., Zhu Q., Burton A.R., Sharma B.R., Qi X., Pelletier S., Vogel P. (2016). NLRC3 is an inhibitory sensor of PI3K-mTOR pathways in cancer. Nature.

[30] Watanabe R., Wei L., Huang J. (2011). mTOR signaling, function, novel inhibitors, and therapeutic targets. J. Nucl. Med..

[31] Dancey J. (2010). mTOR signaling and drug development in cancer. Nat. Rev. Clin. Oncol..

[32] Panwar V., Singh A., Bhatt M., Tonk R.K., Azizov S., Raza A.S., Sengupta S., Kumar D., Garg M. (2023). Multifaceted role of mTOR (mammalian target of rapamycin) signaling pathway in human health and disease. Signal Transduct Target Ther..

[33] Marafie S.K., Al-Mulla F., Abubaker J. (2024). mTOR: Its Critical Role in Metabolic Diseases, Cancer, and the Aging Process. Int. J. Mol. Sci..

[34] Ali E.S., Mitra K., Akter S., Ramproshad S., Mondal B., Khan I.N., Islam M.T., Sharifi-Rad J., Calina D., Cho W.C. (2022). Recent advances and limitations of mTOR inhibitors in the treatment of cancer. Cancer Cell Int..

[35] Mao B., Zhang Q., Ma L., Zhao D.-S., Zhao P., Yan P. (2022). Overview of Research into mTOR Inhibitors. Molecules.

[36] Feldman M.E., Apsel B., Uotila A., Loewith R., Knight Z.A., Ruggero D., Shokat K.M. (2009). Active-site inhibitors of mTOR target rapamycin-resistant outputs of mTORC1 and mTORC2. PLoS Biol..

[37] Thoreen C.C., Kang S.A., Chang J.W., Liu Q., Zhang J., Gao Y., Reichling L.J., Sim T., Sabatini D.M., Gray N.S. (2009). An ATP-competitive Mammalian Target of Rapamycin Inhibitor Reveals Rapamycin-resistant Functions of mTORC1. J. Biol. Chem..

[38] Zheng B., Mao J.-H., Qian L., Zhu H., Gu D.-H., Pan X.-D., Yi F., Ji D.-M. (2015). Pre-clinical evaluation of AZD-2014, a novel mTORC1/2 dual inhibitor, against renal cell carcinoma. Cancer Lett..

[39] Janes M.R., Vu C., Mallya S., Shieh M.P., Limon J.J., Li L.S., Jessen K.A., Martin M.B., Ren P., Lilly M.B. (2013). Efficacy of the investigational mTOR kinase inhibitor MLN0128/INK128 in models of B-cell acute lymphoblastic leukemia. Leukemia.

[40] Bhagwat S.V., Gokhale P.C., Crew A.P., Cooke A., Yao Y., Mantis C., Kahler J., Workman J., Bittner M., Dudkin L. (2011). Preclinical characterization of OSI-027, a potent and selective inhibitor of mTORC1 and mTORC2: Distinct from rapamycin. Mol. Cancer Ther..

[41] Korets S.B., Musa F., Curtin J., Blank S.V., Schneider R.J. (2014). Dual mTORC1/2 inhibition in a preclinical xenograft tumor model of endometrial cancer. Gynecol. Oncol..

[42] Voss M.H., Gordon M.S., Mita M., Rini B., Makker V., Macarulla T., Smith D.C., Cervantes A., Puzanov I., Pili R. (2020). Phase 1 study of mTORC1/2 inhibitor sapanisertib (TAK-228) in advanced solid tumours, with an expansion phase in renal, endometrial or bladder cancer. Br. J. Cancer.

[43] Zhang M., Liu Y., Xiong Z.-Y., Deng Z.-Y., Song H.-L., An Z.-M. (2013). Changes of plasma fibroblast growth factor-21 (FGF-21) in oral glucose tolerance test and effects of metformin on FGF-21 levels in type 2 diabetes mellitus. Endokrynol. Pol..

[44] Subbiah V., Coleman N., Piha-Paul S.A., Tsimberidou A.M., Janku F., Rodon J., Pant S., Dumbrava E.E.I., Fu S., Hong D.S. (2024). Phase I Study of mTORC1/2 Inhibitor Sapanisertib (CB-228/TAK-228) in Combination with Metformin in Patients with mTOR/AKT/PI3K Pathway Alterations and Advanced Solid Malignancies. Cancer Res. Commun..

[45] Jang S.K., Hong S.-E., Lee D.-H., Kim J.-Y., Ye S.-K., Hong J., Park I.-C., Jin H.-O. (2021). Inhibition of mTORC1 through ATF4-induced REDD1 and Sestrin2 expression by Metformin. BMC Cancer.

[46] Melnik B.C., Schmitz G. (2014). Metformin: An Inhibitor of mTORC1 Signaling. J. Endocrinol. Diabetes Obes..

[47] Jhanwar-Uniyal M., Gillick J.L., Neil J., Tobias M., Thwing Z.E., Murali R. (2015). Distinct signaling mechanisms of mTORC1 and mTORC2 in glioblastoma multiforme: A tale of two complexes. Adv. Biol. Regul..

[48] Chen C.Y.-C. (2011). TCM Database@ Taiwan: The world’s largest traditional Chinese medicine database for drug screening in silico. PLoS ONE.

[49] Zeng X., Zhang P., He W., Qin C., Chen S., Tao L., Wang Y., Tan Y., Gao D., Wang B. (2018). NPASS: Natural product activity and species source database for natural product research, discovery and tool development. Nucleic Acids Res..

[50] Ntie-Kang F., Zofou D., Babiaka S.B., Meudom R., Scharfe M., Lifongo L.L., Mbah J.A., Mbaze L.M., Sippl W., Efange S.M.N. (2013). AfroDb: A select highly potent and diverse natural product library from African medicinal plants. PLoS ONE.

[51] Sorokina M., Merseburger P., Rajan K., Yirik M.A., Steinbeck C. (2021). COCONUT online: Collection of open natural products database. J. Cheminformatics.

[52] Yang H., Rudge D.G., Koos J.D., Vaidialingam B., Yang H.J., Pavletich N.P. (2013). mTOR kinase structure, mechanism and regulation. Nature.

[53] Khan A., Shahab M., Nasir F., Waheed Y., Alshammari A., Mohammad A., Zichen G., Li R., Wei D.Q. (2023). Exploring the Traditional Chinese Medicine (TCM) database chemical space to target I7L protease from monkeypox virus using molecular screening and simulation approaches. SAR QSAR Environ. Res..

[54] Chien S.-C., Wu Y.-C., Chen Z.-W., Yang W.-C. (2015). Naturally Occurring Anthraquinones: Chemistry and Therapeutic Potential in Autoimmune Diabetes. Evid.-Based Complement. Altern. Med..

[55] Malik M.S., Alsantali R.I., Jassas R.S., Alsimaree A.A., Syed R., Alsharif M.A., Kalpana K., Morad M., Althagafi I.I., Ahmed S.A. (2021). Journey of anthraquinones as anticancer agents—A systematic review of recent literature. RSC Adv..

[56] Qun T., Zhou T., Hao J., Wang C., Zhang K., Xu J., Wang X., Zhou W. (2023). Antibacterial activities of anthraquinones: Structure–activity relationships and action mechanisms. RSC Med. Chem..

[57] Jasemi S.V., Khazaei H., Morovati M.R., Joshi T., Aneva I.Y., Farzaei M.H., Echeverría J. (2024). Phytochemicals as treatment for allergic asthma: Therapeutic effects and mechanisms of action. Phytomedicine.

[58] Zhao L., Zheng L. (2023). A Review on Bioactive Anthraquinone and Derivatives as the Regulators for ROS. Molecules.

[59] Soto-Blanco B., Mandal S.C., Nayak A.K., Dhara A.K. (2022). Chapter 12—Herbal glycosides in healthcare. Herbal Biomolecules in Healthcare Applications.

[60] Zhou Y., He Y.-J., Wang Z.-J., Hu B.-Y., Xie T.-Z., Xiao X., Zhou Z.-S., Sang X.-Y., Luo X.-D. (2021). A review of plant characteristics, phytochemistry and bioactivities of the genus Glechoma. J. Ethnopharmacol..

[61] Kumar S., Pandey A.K. (2013). Chemistry and Biological Activities of Flavonoids: An Overview. Sci. World J..

[62] Kerhoas L., Aouak D., Cingöz A., Routaboul J.-M., Lepiniec L., Einhorn J., Birlirakis N. (2006). Structural Characterization of the Major Flavonoid Glycosides from Arabidopsis thaliana Seeds. J. Agric. Food Chem..

[63] Vukics V., Guttman A. (2010). Structural characterization of flavonoid glycosides by multi-stage mass spectrometry. Mass Spectrom. Rev..

[64] Kufareva I., Abagyan R. (2012). Methods of protein structure comparison. Methods Mol. Biol..

[65] May A., Zacharias M. (2005). Accounting for global protein deformability during protein-protein and protein-ligand docking. Biochim. Biophys. Acta.

[66] Teilum K., Olsen J.G., Kragelund B.B. (2009). Functional aspects of protein flexibility. Cell Mol. Life Sci..

[67] Verma R., Mitchell-Koch K. (2017). In Silico Studies of Small Molecule Interactions with Enzymes Reveal Aspects of Catalytic Function. Catalysts.

[68] Tuccinardi T. (2021). What is the current value of MM/PBSA and MM/GBSA methods in drug discovery?. Expert Opin. Drug Discov..

[69] Virtanen S.I., Niinivehmas S.P., Pentikainen O.T. (2015). Case-specific performance of MM-PBSA, MM-GBSA, and SIE in virtual screening. J. Mol. Graph Model.

[70] Xu L., Sun H., Li Y., Wang J., Hou T. (2013). Assessing the performance of MM/PBSA and MM/GBSA methods. 3. The impact of force fields and ligand charge models. J. Phys. Chem. B.

[71] Koes D.R., Baumgartner M.P., Camacho C.J. (2013). Lessons Learned in Empirical Scoring with smina from the CSAR 2011 Benchmarking Exercise. J. Chem. Inf. Model..

[72] Ravindranath P.A., Forli S., Goodsell D.S., Olson A.J., Sanner M.F. (2015). AutoDockFR: Advances in Protein-Ligand Docking with Explicitly Specified Binding Site Flexibility. PLOS Comput. Biol..

[73] DeLano W.L. (2002). Pymol: An open-source molecular graphics tool. CCP4 Newsl. Protein Crystallogr..

[74] Bell J., Cao Y., Gunn J.R., Day T., Gallicchio E., Zhou Z., Levyb R., Farid R. (2012). PrimeX and the Schrödinger Computational Chemistry Suite of Programs. https://onlinelibrary.wiley.com/doi/abs/10.1107/97809553602060000864.

[75] Case D.A., Cheatham T.E., Darden T., Gohlke H., Luo R., Merz K.M., Onufriev A., Simmerling C., Wang B., Woods R.J. (2005). The Amber biomolecular simulation programs. J. Comput. Chem..

[76] Pearlman D.A., Case D.A., Caldwell J.W., Ross W.S., Cheatham T.E., DeBolt S., Ferguson D., Seibel G., Kollman P. (1995). AMBER, a package of computer programs for applying molecular mechanics, normal mode analysis, molecular dynamics and free energy calculations to simulate the structural and energetic properties of molecules. Comput. Phys. Commun..

[77] Wang J., Wang W., Kollman P.A., Case D.A. (2001). Antechamber: An accessory software package for molecular mechanical calculations. J. Am. Chem. Soc..

[78] Salomon-Ferrer R., Götz A.W., Poole D., Le Grand S., Walker R.C. (2013). Routine microsecond molecular dynamics simulations with AMBER on GPUs. 2. Explicit solvent particle mesh Ewald. J. Chem. Theory Comput..

[79] Roe D.R., Cheatham III T.E. (2013). PTRAJ and CPPTRAJ: Software for processing and analysis of molecular dynamics trajectory data. J. Chem. Theory Comput..

[80] Chen F., Liu H., Sun H., Pan P., Li Y., Li D., Hou T. (2016). Assessing the performance of the MM/PBSA and MM/GBSA methods. 6. Capability to predict protein–protein binding free energies and re-rank binding poses generated by protein–protein docking. Phys. Chem. Chem. Phys..

[81] Wang N., Zhou K., Liang Z., Sun R., Tang H., Yang Z., Zhao W., Peng Y., Song P., Zheng S. (2023). RapaLink-1 outperforms rapamycin in alleviating allogeneic graft rejection by inhibiting the mTORC1-4E-BP1 pathway in mice. Int. Immunopharmacol..

